# Proportions of Pathogenic Bacteria Isolated from Door Locks and Working Benches in Clinical Laboratory: A Laboratory Based Study

**DOI:** 10.24248/eahrj.v6i1.685

**Published:** 2022-07

**Authors:** Abias Anthon Moshi, Ester Colnel Kyara, Patrick Lucas Mabula, Emmanuel Charles Uroki, Debora Charles Kajeguka, Sixbert Isdory Mkumbaye

**Affiliations:** a Kilimanjaro Christian Medical University College, Department of Microbiology and Immunology, Moshi-Kilimanjaro, Tanzania; b Kilimanjaro Christian Medical Centre, Department of Clinical Laboratory, Moshi-Kilimanjaro, Tanzania

## Abstract

**Background:**

Numerous studies have revealed the association of the door handle and contamination of pathogenic bacteria. Door handles of clinical and research laboratories have higher chances of contamination with pathogenic bacteria during closing and opening with contaminated gloves on, or sometimes after visiting the toilets without the use of disinfectant materials. There is limited epidemiological data regarding bacteria cross contamination of door locks of the Clinical laboratory at Kilimanjaro Christian Medical Centre. This study aimed at providing the proportions of bacteria contaminating medical laboratory doors.

**Methods:**

A cross section laboratory-based study was conducted and it involved collection of swab samples from doors and working benches in the clinical laboratory.

**Results:**

Prevalence of *Staphylococcus aureus, Escherichia coli*, Coagulase Negative *Staphylococcus, Bacillus spp., Pseudomonas aeroginosa* and coliforms were (26%, 22%, 18%, 8%, 4% and 4% respectively.

**Conclusion:**

This study has reported high proportion of pathogenic bacteria. The results entails that, internal and external environments are responsible for laboratory door contamination.

## BACKGROUND

A door lock is a mechanical or electronic fastening device that is released by a physical object (such as; a key, keycard, fingerprint, RFID card, security token, coin, etc.), by supplying secret information (such as a number or letter permutation or password), or by a combination thereof or only being able to be opened from one side such as a door chain.^1^ Numerous studies have revealed the association of the door handle and contamination of pathogenic bacteria.^2,3^ Door handles of clinical and research laboratories have higher chances of contamination with pathogenic bacteria during closing and opening with contaminated gloves on, or sometimes after visiting the toilets without the use of disinfectant materials.^4^ Laboratory workers may acquire infection or be contaminated with these pathogenic bacteria after holding the contaminated door locks.^1,5^ A study conducted in Morocco in 2017 reported that 176(88%) of collected swabs from different hospital surfaces had positive bacterial growth. Gram-negative and positive isolates were 51.5% (101/196) and 48.5% (95/196) respectively. The main isolates being *Enterobacter* (31.6%), *Staphylococcus aureus* (24%), *Pseudomonas aeroginosa* (9.2%) and *Acinetobacter* spp (3.3%).^6^

Another study conducted in Nasarawa State University, Keffi, Nigeria, reported that out of a total of 200 handles sampled, 34 (17%) *E.coli* isolates were identified.^7^ Other studies have also shown that 50% of healthcare associated infections are due to contaminated medical devices.^8,9^ According to a World Health Organization (WHO) report, the incidence of Health Care Associated Infections (HCAI) is still high in many parts of the world including Tanzania.^10^ Overall prevalence of HCAIs in developed countries varies between 5.1% and 11.6%, while the overall prevalence of HCAIs in Tanzania is approximately 14.8% which is high compared to prevalence in developed countries. A study conducted in Altnagelvin hospital showed that among pathogenic bacteria contaminating door frames, *Pseudomonas aeruginosa* was the predominant bacteria species.^11^

Contaminations from door locks or door handles associated infections are infections that an individual can get when they come in contact with the door that is contaminated with such pathogenic bacteria and such infection was not present at the time before contact, and the source of the bacterial contaminants is from the environment.^12,13,14^

In some cases, the microorganism originates from staff's own skin, normal flora becoming opportunistic after touching or doing other procedure that compromise the protective skin barrier.

One of the studies that was conducted in Ghana reported that door handles had the highest isolation^23^ and the highest number of differential isolates were from working surfaces.^7^ Of the total bacterial isolates, 46.14% were pathogenic, with *S. aureus* being the highest (14.42%), while 53.86% were non-pathogenic, made up of 45.2% of *Bacillus spp*.^15^ Other studies conducted in Volta regional hospital in Ghana with an estimate of 218 swab sample taken from door handles and working benches showed that a total of 187 (88.8%) bacterial isolates were obtained from the swabs (*P<.0017*) made up of 55.5% non-pathogenic isolates, 33.3% pathogenic isolates and 14.2% no bacteria growth. There was significant difference between pathogenic isolates and no bacterial growth (*P=.0244*). The largest pathogenic isolates were S. aureus (57.6%) and *E. coli* (39.4%) whilst *Bacillus spp*. was the only identified non-pathogenic isolate.^16^ Another study conducted in Ghana in 2017 had a total of 120 swab samples taken from door handles, stair railings and other points of contact at Tamale Teaching Hospital, Tamale Central Hospital and Tamale West Hospital; a total of 47 (39%) positive *S. aureus* samples were isolated from door handles of the 3 hospitals. These findings are in line with what was reported other studies conducted in Ghana and Nepal.^17,18^ Although staff members might not get infected due to contaminated door handles and working benches, patients with open wounds like burns and scratches may be at high risk to infection.

Bacterial cross-contamination is mostly reported to be high in door handles which are never cleaned with cleaning agents or never cleaned at all.^19^ A study conducted in Japan, described the survival of bacteria under dry conditions, mycobacterium species were detected more than two months after inoculation, since most gram positive and gram negative bacterial can survive for weeks up to months under dry condition.^20^ This fact potentiates regularcleaning and disinfection of working benches and would involve implementing protocol based guidelines for cleaning and decontaminaion.^21^ These procedures must be performed more often during working hours so as to minimise chances of contamination among workers.^22,23^ Failure to perform appropriate disinfection and decontamination can lead to spread of pathogenic bacteria and multidrug resistance organisms.^24^

There is limited epidemiological data regarding bacteria cross contamination of door locks of the Clinical laboratory at Kilimanjaro Christian Medical Centre. This study aimed at providing the proportions of bacteria contaminating medical laboratory doors. The study results will ultimately promote infection prevention and control programs of healthcare associated infections.

## METHODOLOGY

### Study Design, Period and Study Area

A cross section laboratory-based study was conducted and it involved collection of swab samples from doors and working benches in the clinical laboratory. In and out door handles and working benches with higher chance of contamination were considered. Door handles which are not in use or closed doors and working bench with minimal exposure to contamination for a period of more than one month prior to the time of the study were excluded.

The study was conducted from July to August 2020. A total of 34 swab samples were collected; 14 swab samples collected from the inside and outside door locks respectively, and 6 swab samples from the working benches. The study was conducted at Kilimanjaro Christian Medical Centre (KCMC), department of Clinical laboratory located in Moshi, Kilimanjaro, North East Tanzania. KCMC hospital is one of the 4 Zonal consultant hospitals of Tanzania. The hospital is 70Km away from Arusha, situated on the slopes of mount Kilimanjaro (M8JG+2X7 on google map).

### Sample Population and Sampling Method

Samples were collected from clinical laboratory door handles (inside and outside) and working benches. All door handles and sample reception benches and sample processing benches were sampled in the study. Sample size was calculated by using TaroYamane formula [n=N/(1+Ne^2^)]^31^ and a minimum sample size of 25 was estimated. The study collected 34 samples.

### Sample Collection

Sterile cotton swabs were moistured using sterile normal saline and used for sample collection on both external and internal surface of door handles and working benches. All swabs were put into stuart media for transportation to the microbiology laboratory. Samples were given unique identification numbers and were all immediately transported to Microbiology departments for microbiological analysis.

### Culture for Swabbed Doors and Benches in Laboratory Sections

34 swab samples were collected; 14 samples were from the inside and out door locks respectively, making a total of 28 swab samples plus 6 swab samples that were collected from working bench surfaces, making a total of 34 swab samples.

### Bacteria Culture

Bacteria culture was used to isolate specific bacteria. Quantitative culture method using calibrated loop technique was used by streaking on the following media;

**Sheep blood agar (BA):** This was used for growth of both gram positive and gram-negative bacteria. Gram positive bacteria show characteristics of haemolysis of blood and are classified as alpha, beta and gamma haemolysis.

**MacConkey Agar (MCA):** Was used for growth of gram-negative bacteria in order to classify lactose from nonlactose fermenters. All the inoculated plates were incubated in the incubator at 35 to 37°C for 18 to 24 hours. The growth rate on culture plates was quantified as +1, +2, and +3 for the growth on primary, secondary and tertiary streaking on the culture plate respectively.

Biochemical tests were performed in order to properly identify the bacteria to spp levels.

Tests such as; Triple Sugar Iron (TSI), Simon's citrate test, Urease test, Sulphur-Indole-Motility agar (SIM), Catalase test, Coagulase test and Oxidase test were performed according to Clinical Laboratory Standard Institute guidelines-M100 (32).

#### Bacitracin and Optochin

Bacitracin and optochin were used to identify group A and beta haemolytic Streptococci species respectively by assessing the sensitivity or resistance of the two discs. Optochin disc is sensitive to Streptococcus pneumonia, while bacitracin is sensitive to *Streptococcus pyogenes*. Other Streptococcus species such as *S. agalactiae* were identified by using CAMP test and bile esculin was used to identify Enterococcus faecalis *(E. faecalis)*.

#### Novobiocin

This disc was used to differentiate coagulase negative Staphylococcus species. *Staphylococcus epidermidis* is sensitive to novobiocin while *Staphylococcus saprophyticus* is resistant to novobiocin disc.

### Quality Control

Quality control was performed to all biochemical identification test and all media by using ATCC control strains. For performance control, *E. coli* ATCC 25922 (Ref. R4601971. Thermo scientific Lenexa KS 66215 USA), *Pseudomonas aeruginosa* ATCC 27065 (Ref. R4607892. Thermo scientific Lenexa KS 66215 USA) and *S. aureus* ATCC 25923 (Ref. R4609022. Thermo scientific Lenexa KS 66215 USA) strains were used as reference strains. These control Bacteria were inoculated on MCA for lactose and non-lactose fermenters respectively and *S. aureus* onto blood agar. Also, these strains were used in different identification tests such as; catalase test, coagulase test and oxidase test. For sterility control, the media were incubated at 37°C, incubator for 18 to 24 hours to see if the media were contaminated or not.

### Data Management and Data Analysis

Every sample was given its identification number, data was recorded in a logbook before being transferred to excel sheet for coding and cleaning. Coded and cleaned data was then transferred to STATA version 14 for analysis. Tables and figure were used to summarise the results in form of percentage proportions.

### Ethical Considerations

The study was approved by Kilimanjaro Christian Medical University College (KCMUCo) ethical committee with certificate number PG 12/2020., Permission to conduct the study was requested from Kilimanjaro Christian Medical Centre administration via the Head of the Clinical Laboratory department.

## RESULTS

### General Results from the Cultured Samples

From the 34 samples recruited, total isolates were 50; of which 45 were from door lock and 5 from the surface of working benches with some samples having mixed bacteria isolated. The inner door lock surfaces had a total of 18 isolates and 32 isolates were from outer door lock surfaces and working benches (Table 1).

**TABLE 1:** Culture Results for Swabbed Doors and Working Benches in the Laboratory (N=50)

Section/Area	Sample	Out lock (sample A)	In lock (sample B)	Total isolatesn
Sterility	Control 1	-	-	Pass
Performance	Control 2	-	-	Pass
Swab	Control 3	-	-	Pass
Microbiology	S1	S. aureus	No Bacteria growth	1
Chemistry 1	S2	No Bacteria growth	S. aureus, E. coli, CNS	3
Hematology	S3	E. coli	No Bacteria growth	1
Serology	S4	S. aureus, CNS	No Bacteria growth	2
Doctors waiting area door	S5	E. coli, S. aureus, Bacillus spp	Coliforms, S. aureus, Bacillus spp	6
Reception door	S6	CNS	S. aureus	2
Molecula Biology 1	S7	No Bacteria growth	No Bacteria growth	0
Secretary door	S8	S. aureus, E. coli, CNS	-	3
Exit door	S9	E. coli, S. aureus, Citrobacter spp	E.col, CNS	5
Changing room	S10	CNS, Corynebacterium	S. aureus, Citrobacter spp	4
Molecular Biology 2	S11	S. aureus, CNS, Bacillus spp	Enterococci	4
Chemistry 2	S12	No Bacteria Growth	E.coli	1
Board room	S13	E. coli, P. aeroginosa	Coliforms	3
Parasitology	S14	Citrobacter spp	No Bacteria growth	1
Sluice room	S15	E. coli, CNS	P. aeroginosa, E. coli, Bacillus spp, Mould	6
Store 1	S16	S. aureus,	-	1
Store 2	S17	CNS, Corynebacterium	-	2
Store 3	S18	No Bacteria growth	-	0
Working Benches				
Blood transfusion	S19	E. coli, S. aureus corynebacterium	-	3
Reception bench	S20	S. aureus, GPC undetermined	-	2
**Total isolates**		**32**	**18**	**50**

Key: CNS is Coagulase Negative Staphylococcus; GPC is Gram positive cocci; NLF is Non Lactose fermenters 1-6 Number of isolates

### Proportion of Bacteria Isolated from the Door Locks and Working Benches (N=50)

**FIGURE 1:**
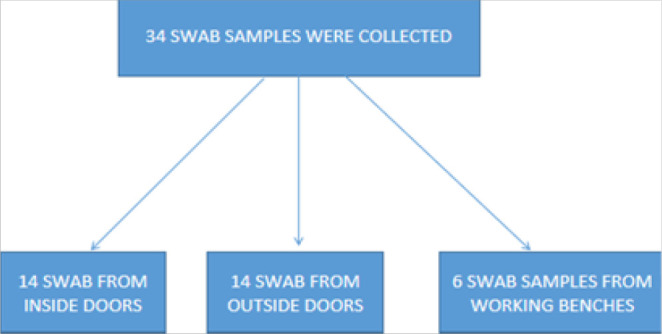
Schematic Presentation of Sampling

The overall door lock swabs were 28 which resulted into 45 isolates. In door locks were highly contaminated by pathogenic *E. coli* (8.0%) and *S. aureus* (12.0%) and out door lock were also highly contaminated with same the pathogenic *E. coli* (8.8%) and *S. aureus* (14.0%), however, Coagulase negative Staphs isolates were high (18.0%) (Table 2). Figure 1

**TABLE 2:** Percentage proportion of the Bacteria Isolated (N=50)

	In lock n (%)	Out lock n (%)		Total N (%)
Total Bacteria growth	9 (26.5)	16 (47.0)		25 (73.5)
Total Negative growth	5 (14.7)	4 (11.8)		9 (26.5)
			**Total**	**34 (100)**
Bacteria isolated				
E. coli	4 (8.0)	6 (12.0)		10 (20.0)
S. aureus	4 (8.0)	7 (14.0)		11 (22.0)
Citrobacter spp	1 (2.0)	2 (4.0)		3 (6.0)
Corynebacterium	0 (0)	2 (4.0)		2 (4.0)
Enterococci	1 (2.0)	0 (0)		1 (2.0)
P. aureginosa	1 (2.0)	1 (2.0)		2 (4.0)
CNS	2 (4.0)	7 (14.0)		9 (18.0)
Coliform	2 (4.0)	0 (0)		2 (4.0)
Bacillus spp	2 (4.0)	2 (4.0)		4 (8.0)
mould	1 (2.0)	0 (0)		1 (2.0)
	Working benches surface	Bench surface		
E. coli	-	1 (2)		1 (2)
S. aureus	-	2 (4)		2 (4)
Corynebacterium	-	1 (2)		1 (2)
GPC undetermined	-	1 (2)		1 (2)
**Total**	**18 (36)**	**32 (64)**		**50 (100.0)**

### Overall Proportions of the Bacteria Isolated

**FIGURE 2:**
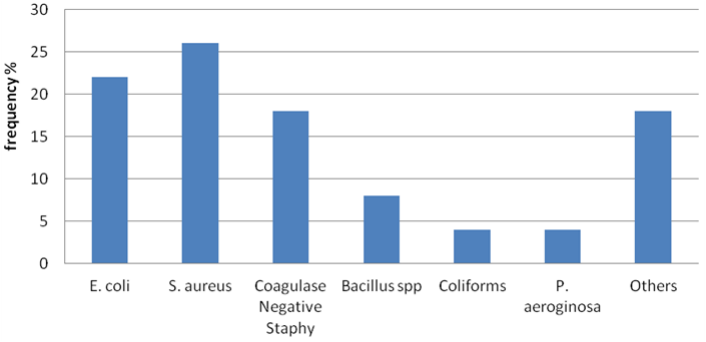
Overall Bacterial Isolates (N=50)

Prevalence of *S. aureus, E. coli*, Coagulase Negative *Staphylococcus, Bacillus spp., P. aeroginosa* and coliforms were (26%, 22%, 18%, 8%, 4% and 4% respectively. Other bacteria isolates and moulds account for 18%, each of which contributing to less than 2% (Figure 2).

## DISCUSSION

The high rate of hospital associated infections are associated with contaminations that occurs as a results of poor hygienic practices and failure to adhere to proper hospital infections control programs.^1^ The high rate of contamination is facilitated by practices such as; inappropriate operation of doors while wearing contaminated gloves, use of toilets without washing hand and non-use of disinfectant material.^3^

This study reported high proportion of bacteria colonising the studied sites (73%). This is high when compared to a study conducted in U. S. A involving elevator buttons of 4 different hospitals which reported the proportion of bacteria colonisation to be 61%.^34^ The high proportion of bacteria colonisation reported in this study can be explained by the fact that, samples in this study were collected from the laboratory area, which is the most contaminated area in the hospital since it is where infectious materials are handled. The study reported high number of Bacteria isolate from the door locks and working benches. The leading bacteria isolates reported are; *S. aureus* followed by *E. coli* and Coagulase Negative Staphylococcus accounting for about 26%, 22% and 18% respectively. These findings are in line with previous studies conducted elsewhere.^5, 25^ However, this study reported slightly higher proportion of *E. coli* isolated from doors compared to a study which was conducted in Nigeria.^33^ The differences in the two study's findings can be accounted for by the fact that, the Nigerian study was conducted in a University premises doors. Such doors are obviously less likely to be as contaminated as Clinical laboratory doors. Hospital environment is highly contaminated and substantially infectious and thus, chances of isolating high number of pathogenic bacteria in such an environment is high.^25,26^ Furthermore, in a study conducted in USA,^5^ the predominant bacteria isolated reported was *S. aureus* and this is in line with this study's results, however, the proportions of the S. aureus in the current study is low compared to the USA study. This might have been attributed by the larger samples size utilised in the USA study.

With the rapid increase of the point prevalence of nosocomial infections among hospital inpatients in developing countries, there is still a big challenge on how best hospital associated infections can be controlled.^27^ Despite the differences in the compared studies' set ups and geographical locations, the colonisation of bacterial in hospital settings is still high and poses a substantially high risk of continued exposure of healthcare workers, patients and patients relatives.^27,28^

This study also reported significant proportion of Coagulase Negative Staphylococci, which is in line with studies conducted in Florida USA, detailing with the fact that; Coagolase negative Staphylococci are colonisers of the skin and are among laboratory contaminants, but are infectious if they penetrate bleached skin.^29,30^ Hospital acquired infections as a result of surface contamination is being implicated in the propagation of drug-resistant bacteria.^13,14^

More interesting in this study, it was found that, more bacteria (CNS, *S. aureus and E. coli*) have been isolated from doors entering Doctor's waiting area before they come into contact with the reception area. This implies that, laboratory premises get contaminated from within source materials as well as from outside, that is to, Laboratory immediate clients who are the Doctors and Nurses during sample receiving process and results issuing.

This is further explained from the observations of our findings: Outside doors were having more bacterial contaminations, this is in line with previous study reports.^17^ This implies that, good hygienic practice is still a problem in hospital settings. With the observed high proportion of pathogenic bacteria isolates. This signifies that, There is need for continued infection and prevention control programs to be in place and operational which goes in line with the COVID 19 era and its recommended control programs.^13^

## CONCLUSION

This study has reported high proportion of pathogenic bacteria. The results entails that, internal and external environments are responsible for laboratory door contamination. Good hygiene practice and infectious control programs should be adhered to and implemented.
